# Exploration of the Tumor Immune Landscape and Identification of Two Novel Immunotherapy-Related Genes for Epstein-Barr virus-associated Gastric Carcinoma *via* Integrated Bioinformatics Analysis

**DOI:** 10.3389/fsurg.2022.898733

**Published:** 2022-05-23

**Authors:** Shi-Zhou Deng, Xiang-Xu Wang, Xing-Yu Zhao, Yin-Miao Bai, Hong-Mei Zhang

**Affiliations:** ^1^ Department of Clinical Oncology, Xijing Hospital, Air Force Medical University, Xi’an, China; ^2^ State Key Laboratory of Cancer Biology, Xijing Hospital of Digestive Diseases, Air Force Medical University, Xi’an, China

**Keywords:** gastric carcinoma, EBVaGC, bioinformatics analysis, tumor microenvironment, biomarker

## Abstract

Epstein–Barr virus (EBV)-associated gastric carcinoma (EBVaGC) is a specific molecular subtype of gastric carcinoma with a high proportion of tumor-infiltrating lymphocytes. It is a highly immunogenic tumor that may benefit from immunotherapy. Hence, it is imperative to analyze the immune landscape and identify immunotherapy biomarkers for EBVaGC. In our study, we investigated the immune landscape and identified 10 hub genes for EBVaGC *via* integrated bioinformatics analysis. We found that EBVaGC expressed more immune-related genes, including common immune checkpoints and human leukocyte antigen (HLA) genes than EBV-negative gastric carcinoma (EBVnGC). The immune score in EBVaGC was higher, which means EBVaGC has greater immune cell infiltration. Ten hub genes (*CD4*, *STAT1*, *FCGR3A*, *IL10*, *C1QA*, *CXCL9*, *CXCL10*, *CXCR6*, *PD-L1*, and *CCL18*) were detected as candidate biomarkers for EBVaGC. Two hub genes, *CXCL9* and *CXCR6*, were identified as novel immunotherapy-related genes. Taken together, the results of our comprehensive analysis of the immune microenvironment of EBVaGC revealed its unique immune landscape, demonstrating that it is a highly immunogenic tumor. Moreover, we identified hub genes that may serve as potential immunotherapy biomarkers for EBVaGC.

## Introduction

Gastric carcinoma (GC) is a common aggressive cancer with increasing incidence and morbidity (1). In 2014, based on the sequencing results of fresh frozen tissues from 295 cases in the cancer genome atlas (TCGA), GC can be divided into four molecular subtypes, including Epstein–Barr virus (EBV), microsatellite instability, genomically stable, and chromosomal instability (2). In 2018, a comparative molecular analysis of 921 cases of gastrointestinal adenocarcinoma was conducted, and a new subtype was added, elevated single nucleotide variant (HM-SNV, *n* = 19) (3). Because of the molecular heterogeneity of GC, the treatment methods and clinical outcomes can vary. Consequently, it is imperative to identify new biomarkers and therapeutic targets to promote individualized treatment for and to improve the management of different subtypes of GC.

EBV-associated gastric carcinoma (EBVaGC) is a unique subtype accounting for approximately 8.7% of GC cases, depending on region and race (4, 5). The occurrence of EBVaGC can be attributed to many factors, including changes in gastric mucosal inflammation, hypermethylation of tumor suppressor genes, and host immune escape driven by EBV infection (5). Previous studies have shown that the molecular signature of EBVaGC is unique. For example, *PD-L1*, also known as CD274, is highly expressed in EBVaGC, which makes EBV a potential biomarker for immunotherapy (2, 6). A phase II clinical trial evaluating the response to PD-1 inhibitor treatment for advanced GC (including six EBVaGC patients) found that all of the patients with EBVaGC achieved a partial response (PR) (7). However, another trial reported that three of four (75%) EBVaGC patients did not achieve PR (8). Interestingly, PD-L1 expression was found to be positive in all of the patients who achieved PR. A study that collected clinical and immunotherapy data on approximately 39 EBVaGC patients from 8 reports concluded that the objective response rate (ORR) was significantly higher in PD-L1-positive EBVaGC patients (63.3%) than in PD-L1-negative EBVaGC patients (0%) after receiving anti-PD-1 antibody monotherapy (9). Although EBV can be a biomarker of immunotherapy for GC to some extent, it also has certain limitations, and more biomarkers are needed to guide the individualized treatment of EBVaGC.

The tumor immune microenvironment is closely related to the progression of GC (10). The immune response, immune cell recruitment, and immune-related molecule regulation can be affected by EBV infection (11). The dysregulation of immune response genes observed in EBVaGC patients is conducive to recruiting more reactive immune cells (12). Typical lymphoepithelioma-like GC and Crohn’s disease-like lymphocytic reaction, two subtypes of EBVaGC with a high degree of lymphocytic infiltration, account for more than 85% of EBVaGC (13). The tumor immune microenvironment of EBVaGC has extensive immune cell infiltration, with the highest proportion of these cells being CD8+ T cells (14).

We performed a comprehensive analysis of the immune microenvironment of EBVaGC, which revealed differences in the immune landscape of the tumor microenvironment (TME) and the expression of common immune checkpoints (ICPs) and HLA genes among EBVaGC and EBV-negative gastric carcinoma (EBVnGC). Furthermore, we also used multiple data sets to predict the efficacy of immunotherapy for EBVaGC. Additionally, through a series of algorithms, we identified EBVaGC-specific immune-related genes (IRGs) and hub genes and carried out validation in an external database. To our knowledge, it is the first time to make an analysis of the immune microenvironment of EBVaGC *via* integrated bioinformatics analysis. Our findings will improve our understanding about special features of EBVaGC, it may provide a new idea of the occurrence and development mechanism and provide potential immunotherapy biomarkers for EBVaGC.

## Materials and Methods

### EBVaGC and EBVnGC Data Sets and Immune-Related Gene Sources

RNA-seq and survival data of EBVaGC and EBVnGC were extracted from TCGA (https://portal.gdc.cancer.gov/) data sets. Microarray data (Affymetrix) were extracted from Gene Expression Omnibus (GEO, https://www.ncbi.nlm.nih.gov/geo/) datasets. A total of 401 gastric carcinoma samples were included in this study, including 375 from TCGA-STAD (EBVaGC = 27, EBVnGC = 348) data sets and 26 from the GSE51575 cohort (EBVaGC = 12, EBVnGC = 14). IRGs were obtained from InnateDB data sets (15) (https://innatedb.com/index.jsp) and ImmPort data sets (16) (https://www.immport.org/home).

### Enrichment Analysis of EBVaGC-Related Differentially Expressed Genes

Firstly, we identified differentially expressed genes (DEGs) and differentially expressed immune-related genes (DE-IRGs) (adjusted *p* < 0.05, absolute value of Log2 FC ≥1) between EBVaGC and EBVnGC using the R package “limma” (17). Then, the R packages “clusterProfiler”, Gene Ontology (GO), and Kyoto Encyclopedia of Genes and Genomes (KEGG) were utilized to uncover the biological functions and associated signaling pathways (18). Thirdly, in the Molecular Signatures Database, we downloaded C2 collection (KEGG gene sets) and Hallmark gene sets, which were analyzed through GSEA software (Broad institute, USA). The selection criteria for significant gene sets were *p* < 0.05 and False Discovery Rate (FDR) *q*-value < 0.25.

### Comparison of the Tumor Microenvironment of EBVaGC and EBVnGC

Single-sample gene-set enrichment analysis (ssGSEA) was utilized to quantify the enrichment levels of the 29 immunological signatures in each GC sample (19). The stromal score, immune score, ESTIMATE score, and tumor purity were estimated by the “ESTIMATE” package (20). The fractions of 22 human immune cell subsets in GC samples were calculated with the deconvolution approach CIBERSORT. The main criteria for sample selection were CIBERSORT-*p *< 0.05. Next, we compared the fractions about these 22 human immune cell subsets among EBVaGC and EBVnGC by the MannWhitney *U* test.

### Identification of EBVaGC-Specific Immune-Related Genes and HUB Genes

After identifying DE-IRGs, we then used weighted gene coexpression network analysis (WGCNA) (21) and found a gene module related to EBVaGC. Next, based on the STRING database version 11.0 (22), we performed protein–protein association analysis of candidate genes and excluded genes that had no predicted interaction with the other candidate genes. Additionally, utilizing Cytoscape software and its plugin, CytoHubba, we constructed a protein–protein interaction (PPI) network and explored 10 PPI network HUB genes ranked by the betweenness method.

### Cell Lines

AGS and SNU-719 were human EBVnGC and EBVaGC cell line, respectively. The culture medium of these two cell lines were RPMI-1640 medium (Gibco, USA), which was supplemented with 2% penicillinstreptomycin and 10% fetal bovine serum (incellgene). The culture environment: 37 °C and 5% CO_2_.

### Reverse Transcription-Polymerase Chain Reaction

TRIzol reagent (Invitrogen, China) was applied to extract total RNA. The Evo M-MLV RT Kit (Accurate Biology, China) was used to carry out Reverse transcription.

The following sequences are the primers of CXCL9 and CXCR6:

CXCL9:

F (5′–3′) CAGTAGTGAGAAAGGGTCGC
R (5′–3′) AGGGCTTGGGGCAAATTGTT

CXCR6:

F (5′–3′) GACTATGGGTTCAGCAGTTTCA
R (5′–3′) GGCTCTGCAACTTATGGTAGAAG

Quantitative reverse transcription-polymerase chain reaction (qRT-PCR) was carried out by the SYBR Green Premix Pro Taq HS qPCR Kit (Accurate Biology, China). The above operations were performed followed the instructions of manufacturer.

### Survival Analyses

After excluding data from patients who survived less than 90 days, 321 patients were included in the survival analysis. Used the “surv_cutpoint” function in “survminer” package, we computed the optimal cutoff point of the hub genes and draw KaplanMeier survival curves. The survival difference of each group was evaluated by log-rank test.

## Results

### Identification of EBVaGC-Related Differentially Expressed Genes in the TCGA Cohort

**Figure 1:**
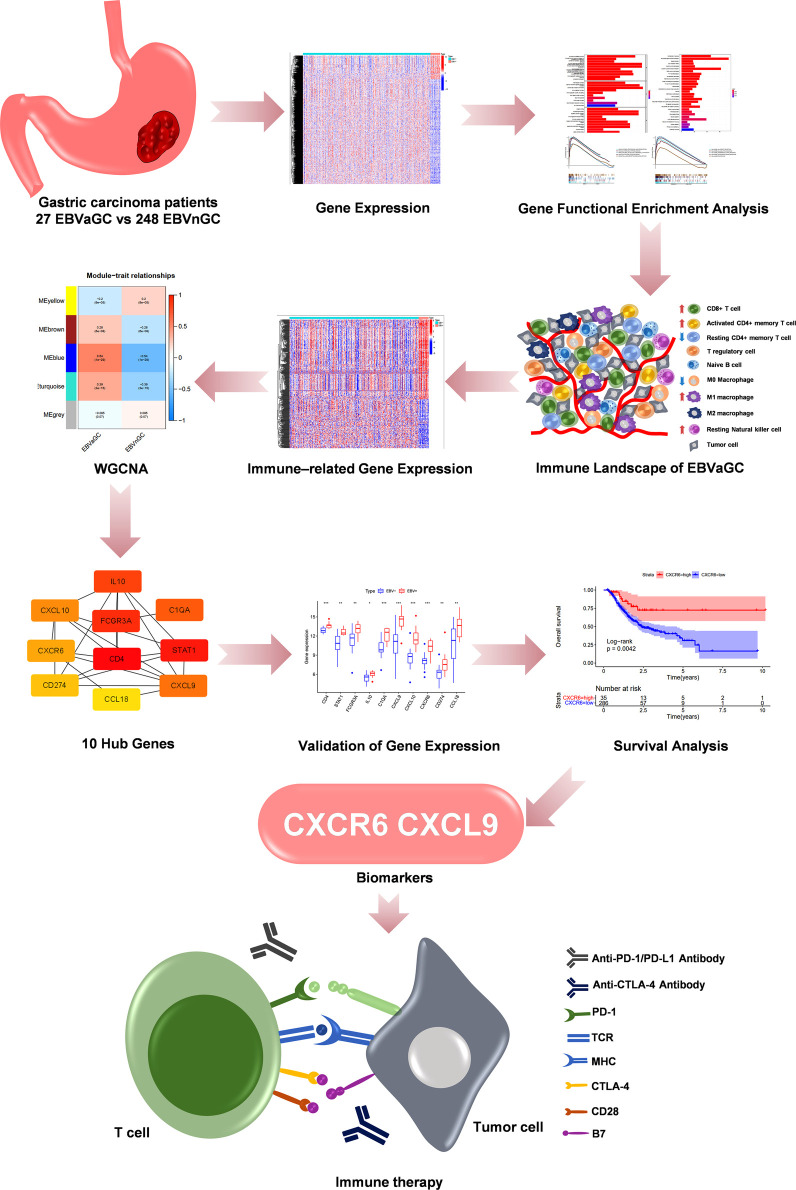
The flow diagram and analysis methods of our study.

**Figure 2:**
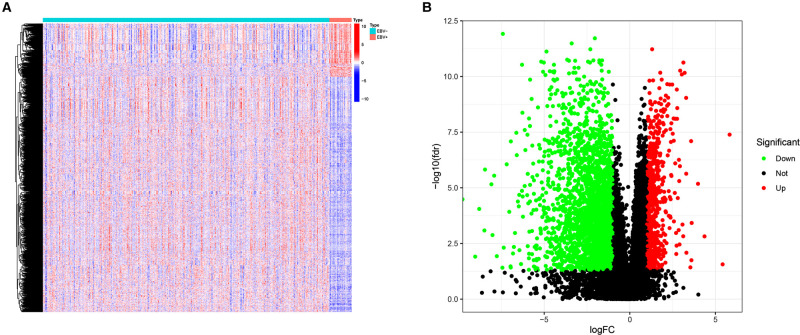
DEGs. (**A**) Heatmap for DEGs between EBVaGC(EBV+) and EBVnGC(EBV−). (**B**) Volcano plot of DEGs between EBVaGC and EBVnGC.

Figure 1 shows the flow diagram of our study. A total of 375 gastric carcinoma patients (including 27 EBVaGC and 248 EBVnGC) from TCGA were used as a discovery cohort. The baseline profiles of the patients are listed in Table 1. To evaluate the DEGs between EBVaGC and EBVnGC, we analyzed RNA-seq data and identified 3,584 EBVaGC-related DEGs, of which 2,912 were downregulated and 672 were upregulated (Figures 2A,B).

**Table 1:** Clinical characteristics of EBVaGC and EBVnGC patients in TCGA.

	EBVaGC	EBVnGC
No. of patients	27	348
Age		
≤65	14	150
>65	13	194
Unknown	0	4
Gender		
Female	4	130
Male	23	218
Grade		
G1	0	10
G2	2	135
G3	25	194
GX	0	9
Stage		
I	1	52
II	7	104
III	17	133
IV	2	36
Unknown	0	23
Survival status		
OS day (median)	593	426
Ending		
Survival	17	211
Death	10	137

*EBVaGC, Epstein–Barr virus-associated gastric carcinoma; EBVnGC, EBV-negative gastric carcinoma.*

### Enrichment Analysis of EBVaGC-Related Differentially Expressed Genes

**Figure 3:**
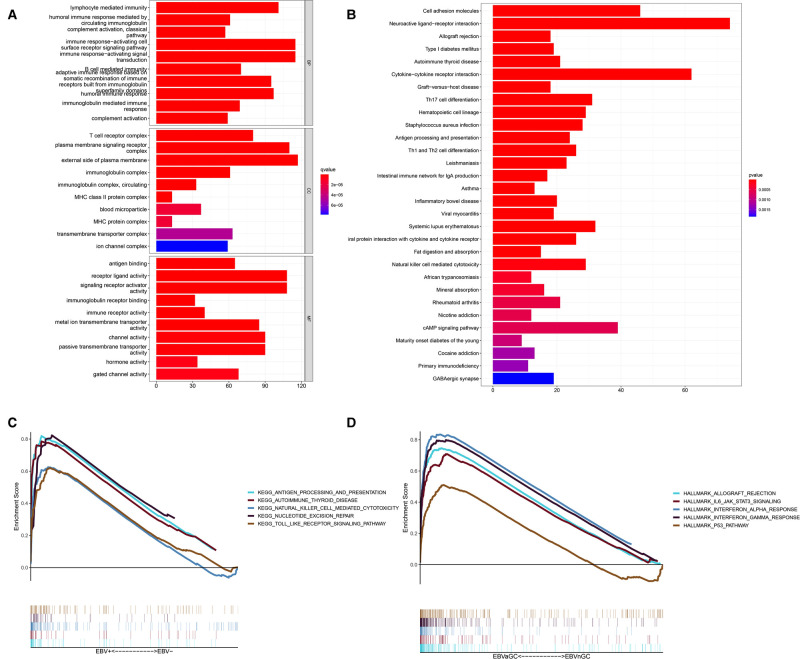
DEGs and functional enrichment analyses. (**A**) GO analysis. (**B**) KEGG pathway analysis. (**C,D**) GSEA for EBVaGC samples. Enriched gene sets in C2 collection, the KEGG gene sets (**C**) and HALLMARK collection (**D**).

Next, we performed enrichment analyses of the DEGs. GO analysis showed that the DEGs were involved in biological processes related to immunity, including lymphocyte-mediated immunity, the humoral immune response mediated by circulating immunoglobulin, the classical complement activation pathway, etc. (Figures 3A). The results of KEGG analysis were consistent with the results of GO analysis, revealing DEGs mainly involved in Th17-cell differentiation, antigen processing, Th1 and Th2 cell differentiation and antigen presentation, and other immune-related pathways (Figure 3B). GSEA was also conducted in the EBVaGC and EBVnGC groups. For the C2 collection (KEGG gene sets), the top five pathways with enrichment in the EBVaGC group were highly correlated with immune biological processes such as antigen processing and presentation and natural killer (NK) cell-mediated cytotoxicity (Figure 3C). Furthermore, the results for HALLMARK gene sets illustrated immune activities, including allograft rejection, interferon-alpha response, and interferon-gamma response, were involved in EBVaGC (Figure 3D).

### Different Immune Landscapes of the Tumor Microenvironment between EBVaGC and EBVnGC

**Figure 4:**
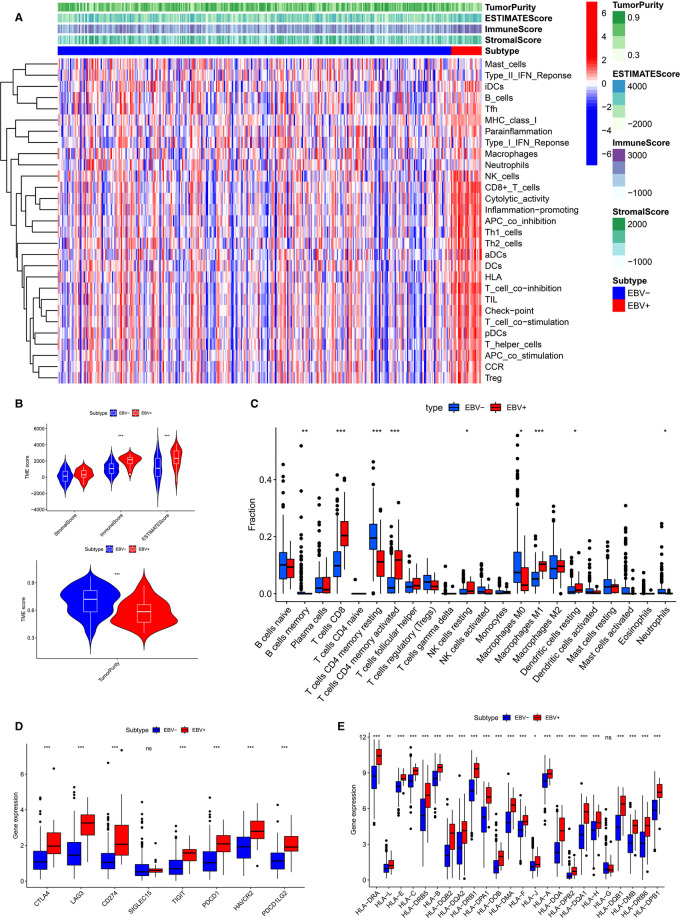
Differences of TME characteristics between EBVaGC and EBVnGC. (**A**) ssGSEA analysis of EBVaGC (red) and EBVnGC (blue) samples according to 29 immune-associated gene sets. ESTIMATE was used to evaluate stromal score, immune score, ESTIMATE score, and tumor purity. (**B**) Differences of stromal score (*p* > 0.05), immune score (*p* < 0.05), ESTIMATE score (*p* < 0.05), tumor purity (*p* < 0.05). (**C**) Differences in the proportion of immune cell infiltration analyzed by CIBERSORT. (**D,E**) The expression of ICPs and HLA genes expression levels between EBVaGC and EBVnGC. CD274 (PD-L1), PDCD1(PD-1), PDCD1LG2 (PD-L2).

The results of the enrichment analysis revealed differences in immune-related functions and pathways between EBVaGC and EBVnGC. We applied ssGSEA, ESTIMATE, and CIBERSORT to further investigate these differences in terms of the immune landscape of the TME. First, we employed ssGSEA to examine transcriptome data from GC tissue samples. According to the heatmap, the EBVaGC group expressed more IRGs than the EBVnGC group (Figure 4A). ESTIMATE analysis showed that the ESTIMATE score and immune score were higher in the EBVaGC subgroup and that the tumor purity of EBVaGC was lower (Figure 4B). Twenty-two types of tumor-infiltrating immune cell (TIC) profiles were constructed from the GC samples, and the proportion of TIC subtypes in EBVaGC and EBVnGC is shown in Supplementary Figure S1A. We also tested the correlation of these TICs to further investigate their characteristics in the TME (Supplementary Figure S1B). The proportions of nine kinds of TICs were found to be different between EBVaGC and EBVnGC (Figure 4C). There was a higher abundance of CD8 T cells, CD4 activated memory T cells, resting NK cells, M1 macrophages, and resting dendritic cells and a lower abundance of memory B cells, resting CD4 memory T cells, M0 macrophages, and neutrophils in EBVaGC. Moreover, we can see that the highest proportion of TICs in EBVaGC is CD8 T cells. The above results indicate that the immune landscape of the TME was significantly different between EBVaGC and EBVnGC. EBV infection may affect the TME status of GC and could explain why EBVaGC is a highly immunogenic tumor.

### Expression of Immune Checkpoint and Human Leukocyte Antigen Genes among EBVaGC and EBVnGC

Next, we investigated the difference between EBVaGC and EBVnGC in the expression of common ICPs and HLA genes (Figures 4D,E). The ICPs include Siglec-15, CTLA4, LAG3, PD-1, PD-L1, TIGIT, PD-L2, and HAVCR2. Siglecs are novel promising targets for GC immunotherapy, among which Siglec-15 has been widely studied (23). A previous study has explored the expression of Siglec-15 in GC tissues and evaluated its clinical value (24). In our study we found that the Siglec-15 expression between EBVaGC and EBVnGC has no marked difference (Figure 4D). However, the levels of CTLA4, PD-1, PD-L1, LAG3, TIGIT, HAVCR2 and PD-L2 were higher in EBVaGC than in EBVnGC (Figure 4D). This means that GC patients infected with EBV may be more sensitive to immunotherapy treatment. The expression of most HLA genes, except HLA-G, was higher in EBVaGC (Figure 4E). These results revealed that the EBV infection status significantly impacts the expression of ICPs and HLA genes in GC.

### Differentially Expressed Immune-Related Genes and HUB Genes

**Figure 5:**
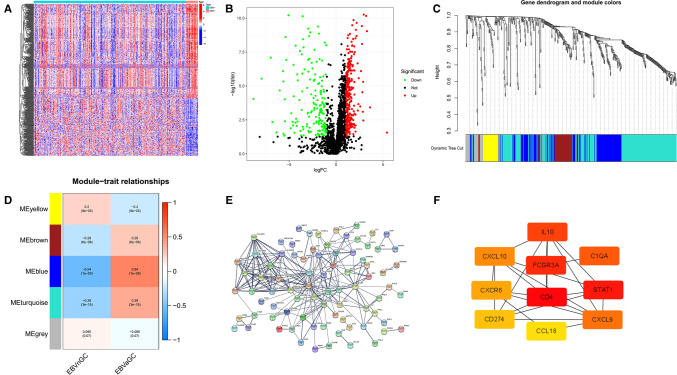
DE-IRGs and HUB genes. (**A**) Heatmap for DE-IRGs among EBVaGC and EBVnGC. (**B**) Volcano plot for DE-IRGs between EBVaGC and EBVnGC. (**C**) Identification of coexpression modules in EBVaGC. The cluster dendrogram’s branches reflect the five distinct gene modules. Each module represents a group of corelated genes and has its own color. Each leaf segment on the cluster dendrogram represents a gene. (**D**) Heatmap illustrates the relationship between gene modules and EBVaGC or EBVnGC. (**E**) PPI network of genes in the blue module created by STRING. (**F**) Ten HUB genes identified by Cytoscape plugin CytoHubba. The color of the nodes reflects the degree of connection; red represents a higher degree, while yellow represents a lower degree.

**Figure 6:**
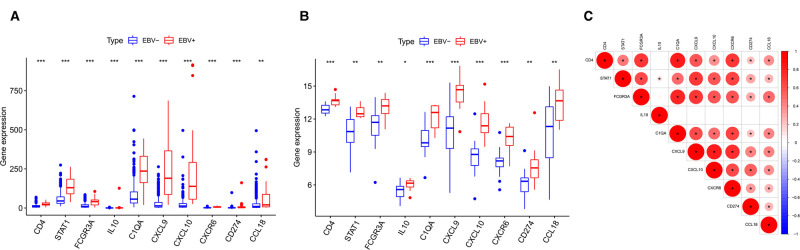
Differences in HUB gene expression levels. (**A**) Relative expression levels of HUB genes in the TCGA cohort. (**B**) Validation in the GEO cohort. (**C**) Correlation between the HUB genes.

According to the above analysis results, we identified DE-IRGs between EBVaGC and EBVnGC. The volcano map and heatmap show the expression profiles of the 288 upregulated and 169 downregulated genes in EBVaGC (Figures 5A,B). Using the expression matrix of DE-IRGs, we then constructed a scale-free coexpression network by WGCNA. A total of five modules were identified, and we found that the blue module (|*r*| = 0.54, *p* = 1e−29) had the highest association with EBVaGC and regarded these genes as EBVaGC-specific immune-related genes (IRGs) (Figures 5C,D). After uploading the EBVaGC-specific IRGs to the STRING database, we then input the node pairs into Cytoscape software (Institute of Systems Biology, UC San Diego, etc, USA) and visualized genes in the PPI network (Figure 5E). *Via* the Cytoscape plugin CytoHubba, the top 10 hub genes (*CD4*, *STAT1*, *FCGR3A*, *IL10*, *C1QA*, *CXCL9*, *CXCL10*, *CXCR6*, *PD-L1*, and *CCL18*) were identified (Figure 4F) and analyzed further to determine whether they could be considered candidate biomarkers for EBVaGC. We investigated the expression of these 10 hub genes and found that all of them were higher in EBVaGC than in EBVnGC in both the TCGA-STAD cohort and GEO cohort (Figures 6A,B). Figure 6C show the relationship between these 10 hub genes in TCGA.

### Prognostic Significance of *HUB* Genes

**Figure 7:**
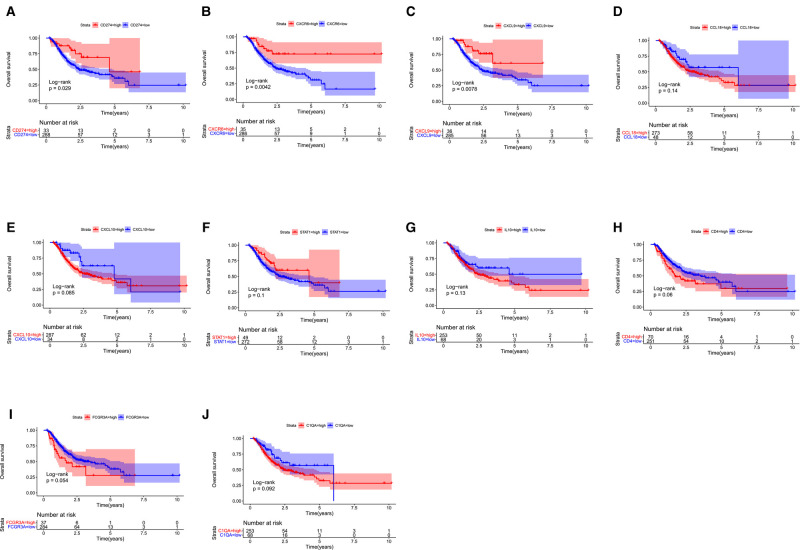
Survival analysis. (**A**–**J**) Kaplan–Meier curve illustrating the prognostic values of 10 HUB genes [CD274(PD-L1), CXCR6, CXCL9, CCL18, CXCL10, STAT1, IL10, CD4, FCGR3A, C1QA].

Next, we probed the prognostic significance of the 10 hub genes in the TCGA-STAD cohort (Figures 7A–J). Patients were classified into high- and low-expression subgroups based on the optimal cutoff point. The Kaplan–Meier curve showed that patients with higher PD-L1, CXCL9, and CXCR6 expression had better overall survival (OS). The expression of the rest of the seven hub genes had no significant correlation with the survival of GC patients. Previous studies (2, 6, 9) have shown that the expression of PD-L1, which is a recognized biomarker for immunotherapy, is higher in EBVaGC than in other types of GC. Therefore, CXCL9 and CXCR6 are selected as candidate biomarkers for EBVaGC and will be the focus of our subsequent study.

### Correlation analysis of HUB Gene Expression with Tumor-Infiltrating Immune Cells and Immune Checkpoints

**Figure 8:**
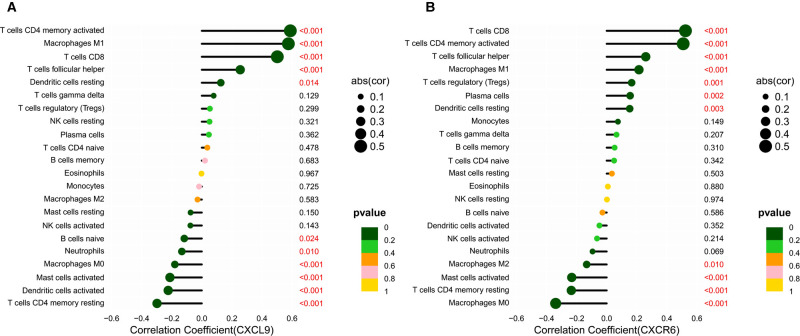
Correlation between the HUB genes with TICs. (**A,B**) Lollipop plots showed correlation of CXCR6 and CXCL9 expression with 22 types of TICs. The size of the lollipop illustrates the level of correlation, and the color of the lollipop indicates the *p*-value.

**Figure 9:**
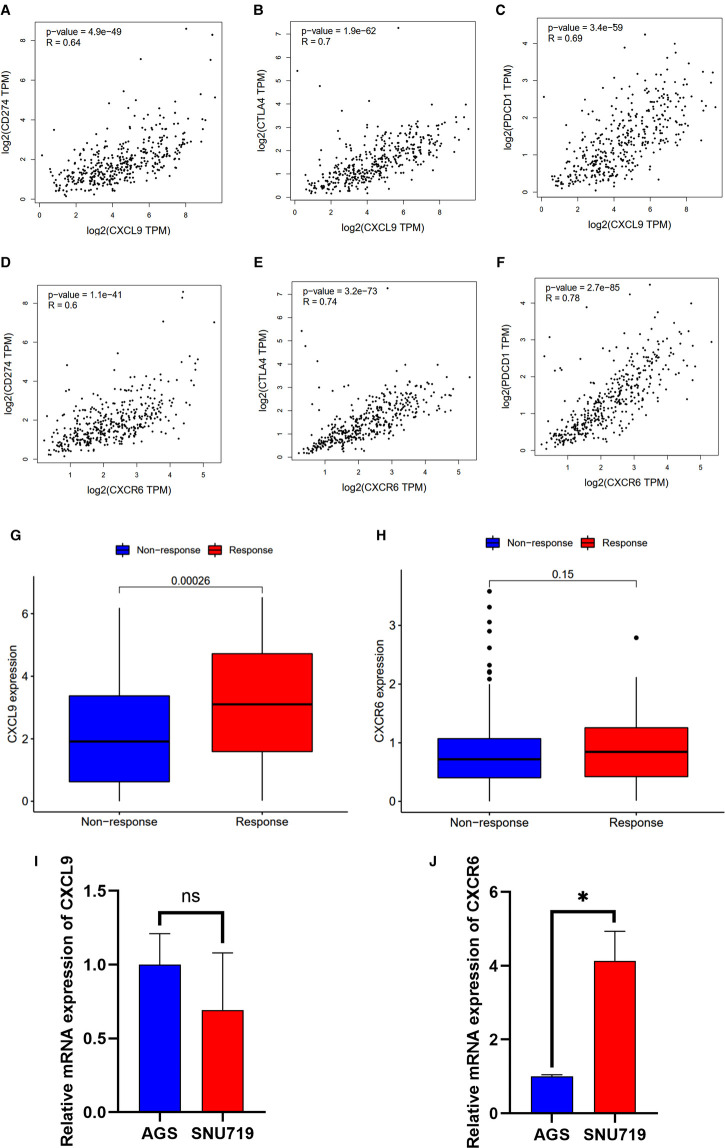
Correlation between the HUB genes with immunotherapy. (**A–F**) Scatter plot showing the correlation of ICPs, CD274(PD-L1), CTLA-4, and PDCD1(PD-1), with the CXCR6 (**A–C**) and CXCL9 (**D–F**) expression (correlation test: Pearson coefficient). (**G,H**) CXCR6 and CXCL9 expression with the effect of immunotherapy. (**I,J**) Validation of mRNA expression of CXCL9 and CXCR6 in human EBV-associated gastric cell lines (SNU-719, red) and EBV-negative GC cell lines (AGS, blue).

Based on the CIBERSORT results of GC, we investigated the correlation between the expression of the hub genes and 22 kinds of TICs (Figure 8A,B, Supplementary Figures S2A–H). CXCL9 expression was positively related to five TICs, namely, CD4 activated memory T cells, macrophage M1, CD8 T cells, follicular helper T cells, and resting dendritic cells, and was negatively related to six TICs, namely, CD4 resting memory T cells, activated dendritic cells, activated mast cells, macrophage M0, neutrophils, and naive B cells (Figure 8A). The correlation between the expression of CXCR6 and the remaining eight hub genes with TICs is shown in Figure 8B and Supplementary Figure S2. Furthermore, we revealed the correlations between CXCL9/CXCR6 expression and multiple ICPs by Gene Expression Profiling Interactive Analysis (25) (Figures 9A–F). CXCL9 and CXCR6 have a positive relationship with three common ICPs: PD-L1, CTLA-4, and PD-1. IMvigor210, a urothelial cancer dataset receiving anti-PD-L1 therapy, was utilized to predict the benefit of immunotherapy (26). CXCL9 expression was higher in the response group than in the no-response group (Figure 9G). Although CXCR6 expression levels did not differ between the response and no-response groups (*p* = 0.15) (Figure 9H), we could still see that CXCR6 expression in the response group showed an upward trend. These results suggested that CXCL9 and CXCR6 may serve as immunotherapy biomarkers for EBVaGC. The RNA expression of CXCL9 and CXCR6 in human EBV-associated gastric cell lines (SNU-719) and EBV-negative GC cell lines (AGS) were evaluated by qRT-PCR (Figures 9I,J). The expression of CXCR6 was higher in SNU-719, while the CXCL9 expression between SNU-719 and AGS has no marked difference.

## Discussion

Because of the heterogeneity of GC, the characteristics of the different subtypes must be investigated so that new and subtype-specific biomarkers can be identified to improve precision medicine. Among all subtypes of gastric carcinoma, patients with EBVaGC have better OS (27). Infection with EBV leads to an activated immune response, immune cell recruitment, and immune-related molecule regulation (11). Further elucidation of the differences between EBVaGC and EBVnGC in terms of the tumor immune microenvironment by bioinformatics analysis will help deepen our understanding of the characteristics of EBVaGC. Although patients with EBVaGC have an immunotherapeutic advantage in theory, there are still some patients who are insensitive to PD-1/PD-L1 inhibitor monotherapy (9). After receiving PD-1 inhibitor monotherapy, the ORR in PD-L1-positive EBVaGC patients was approximately 63.3%. Therefore, other biomarkers must be identified in addition to PD-L1 to guide the immunotherapy of EBVaGC.

In our study, we analyzed the DEGs between EBVaGC and EBVnGC in the TCGA database. Then, GO and KEGG enrichment analyses were performed based on these DEGs, and the results revealed that they were closely related to immune-related biological processes and immune-related pathways. GSEA, a typical method for integrating gene expression information, can directly determine the expression trends of gene sets in different groups (28). Hence, we conducted GSEA to further investigate the differences between EBVaGC and EBVnGC and found that the gene sets in the EBVaGC group were highly correlated with immune biological processes. Combining the evidence from an abundance of previous studies with the above results, we speculated that the TME in EBVaGC may be different from that in EBVnGC. Therefore, we explored the immune landscape of GC using different algorithms. The results indicated that the EBVaGC subgroup had a higher abundance of CD8 T cells, CD4 activated memory T cells, resting NK cells, M1 macrophages, M0 macrophages, and the like. The deregulation of immune response genes in EBVaGC is conducive to recruiting more reactive immune cells (12), which also explains the high proportion of immune cells in EBVaGC. Studies have shown that the main TICs of EBVaGC are CD8+ T cells (14). We also observed that the highest proportion of TICs in EBVaGC was CD8+ T cells. On the other hand, the immune score was higher in the EBVaGC subgroup, while the tumor purity was lower.

The TME of gastric carcinoma is affected by multiple genes. Based on the IRGs obtained from the InnateDB and ImmPort immune gene datasets, we identified DE-IRGs in EBVaGC. WGCNA was used to identify DE-IRGs that are closely related to EBVaGC. A total of 122 DE-IRGs were included in the blue module, which can also be called EBVaGC-specific IRGs. Next, we constructed a PPI network and explored 10 hub genes (*CD4*, *STAT1*, *FCGR3A*, *IL10*, *C1QA*, *CXCL9*, *CXCL10*, *CXCR6*, *PD-L1*, and *CCL18*), and the results indicated that they could serve as candidate biomarkers or therapeutic targets. In the GEO cohort, these 10 hub genes were also highly expressed in EBVaGC.

Survival analysis showed that patients with higher CXCR6, CXCL9, and PD-L1 expression had longer OS. PD-L1 is a recognized biomarker for immunotherapy, and its expression was found to be higher in EBVaGC patients. CXCL9 is a member of the chemokine superfamily; it enhances T-cell infiltration and localizes activated T cells near the antigen-presenting cell. CXCL9 is a ligand for CXCR3, along with CXCL10 and CXCL11 (29, 30). CXCL9, CXCL10, and CXCL11/CXCR3 axes can induce immune activation and then inhibit tumor growth, which are potential novel therapeutic targets for cancer therapy (31). CXCL9-expressing tumor-associated macrophages can recruit a large number of CXCR3-expressing stem-like CD8+ T cells into TME, thus improving the efficacy of anti-PD(L)-1 treatment (29). Clinical examination of gastric carcinoma samples revealed that CXCL9 was substantially expressed in highly immunogenic tumors and associated with favorable survival outcomes (32). PD-L1 was elevated in GC cells and tissues following CXCL9/10/11 treatment, which was associated with STAT3 activation (33). CXCR6 is a CXC chemokine receptor, and chemokine ligand 16 is its sole ligand (34). CXCR6 is an important factor in the antitumor activity of CD8 positive T cells in TME (35). CXCR6 may inhibit the occurrence of hepatocellular carcinoma by mediating the clearance of senescent hepatocytes by NKT cells and CD4+ T cells (36). In head, neck, and lung tumors and ovarian cancer, CXCR6 can promote the recruitment of CD8+ T cells in TME (37, 38). CXCL16 overexpression has been shown to activate mitogen-activated protein kinase pathways and elevate CXCR6 expression in GC (39). CXCR6 was found to be dramatically increased in GC tissues, and higher CXCR6 was linked to worse survival outcomes (40). This result seems inconsistent with our study. A recently published study showed that CXCR6 is essential for sustained tumor control mediated by CD8+ cytotoxic T cells (CTLs) and improves TCF-1neg CTL antitumor activity in the TME (41). This confirms our conclusion that CXCR6 is a candidate biomarker for immunotherapy. The results regarding the relationship of HUB genes with TICs and ICPs demonstrate that CXCL9 and CXCR6 are highly related to macrophage M1 and CD8 T cells, respectively. CXCL9 and CXCR6 were also found to be positively related to three ICPs, PD-L1, CTLA-4, and PD-1. In the absence of publicly available data on immunotherapy for GC, we used IMvigor210 to predict the anti-PD-L1 immunotherapy response. We can conclude that CXCL9 and CXCR6, together with PD-L1, can serve as immunotherapy biomarkers for EBVaGC based on the expression trends between the Response group and the No-Response group. However, the cell experiments showed that there was no significant difference in the expression of CXCL9 between SNU-719 and AGS, which was inconsistent with the conclusion in the database. The reasons may be as follows: first, we only used EBV-associated gastric cell line (SNU-719) and EBV-negative GC cell line (AGS) to verify CXCL9 expression; more cell lines are needed to corroborate our conclusion; second, CXCL9 is mainly expressed in immune cells, which may also be the cause of the inconsistency between cell experiments and tissue RNA sequencing results in the database.

The main limitation of our study is the lack of sufficient data. First, there were only 27 EBVaGC samples in the TCGA cohort, so we had to use all gastric carcinoma data for survival analysis. Second, in the absence of public data on immunotherapy for gastric carcinoma, we cannot use other data (IMvigor210) to predict immunotherapy efficacy. However, even when using alternative data for analysis, our conclusions remain reliable. In addition, due to the low incidence of EBVaGC, it is difficult to collect clinical samples to validate our studies.

The potential application of PD-1/PD-L1 inhibitor monotherapy and combination therapy provides new opportunities for EBVaGC patient management. Screening suitable populations for immunotherapy through biomarkers is one way to realize precision medicine and individualized treatment. CXCL9 and CXCR6, like PD-L1, have the potential to serve as immunotherapy biomarkers for EBVaGC. Further experimental and clinical studies are necessary to verify these conclusions.

## Data Availability

The datasets presented in this study can be found in online repositories. The names of the repository/repositories and accession number(s) can be found in the article/Supplementary material.
