# Supplementary material for: Exploration of the Tumor Immune Landscape and Identification of Two Novel Immunotherapy-Related Genes for Epstein-Barr virus-associated Gastric Carcinoma via Integrated Bioinformatics Analysis
Source: Front Surg. 2022 May 23;9:898733. doi: 10.3389/fsurg.2022.898733 (PMC9450882; doi:10.3389/fsurg.2022.898733)
Supplement: Supplementary file 1 [file Data_Sheet_1_v1.pdf]

Editing Certificate

This document certifies that the manuscript

Immune Landscape and Identification of two novel immunotherapy-related genes for EBV-associated GC Via Integrated Bioinformatics Analysis

prepared by the authors

Shi-Zhou Deng1#, Xiang-Xu Wang1#, Xing-Yu Zhao2, Yin-Miao Bai1, and Hong-Mei Zhang1*

was edited for proper English language, grammar, punctuation, spelling, and overall style by one or more of the highly qualified native English speaking editors at AJE.

This certificate was issued on **April 20, 2022** and may be verified on the AJE website using the verification code **0DC7-8EEA-4853-6312-106P** .

Neither the research content nor the authors' intentions were altered in any way during the editing process. Documents receiving this certification should be English-ready for publication; however, the author has the ability to accept or reject our suggestions and changes. To verify the final AJE edited version, please visit our verification page at aje.com/certificate. If you have any questions or concerns about this edited document, please contact AJE at support@aje.com.

AJE provides a range of editing, translation, and manuscript services for researchers and publishers around the world. For more information about our company, services, and partner discounts, please visit aje.com.
