# Supplementary figures and images for: Exploration of the Tumor Immune Landscape and Identification of Two Novel Immunotherapy-Related Genes for Epstein-Barr virus-associated Gastric Carcinoma via Integrated Bioinformatics Analysis
Source: Front Surg. 2022 May 23;9:898733. doi: 10.3389/fsurg.2022.898733 (PMC9450882; doi:10.3389/fsurg.2022.898733)

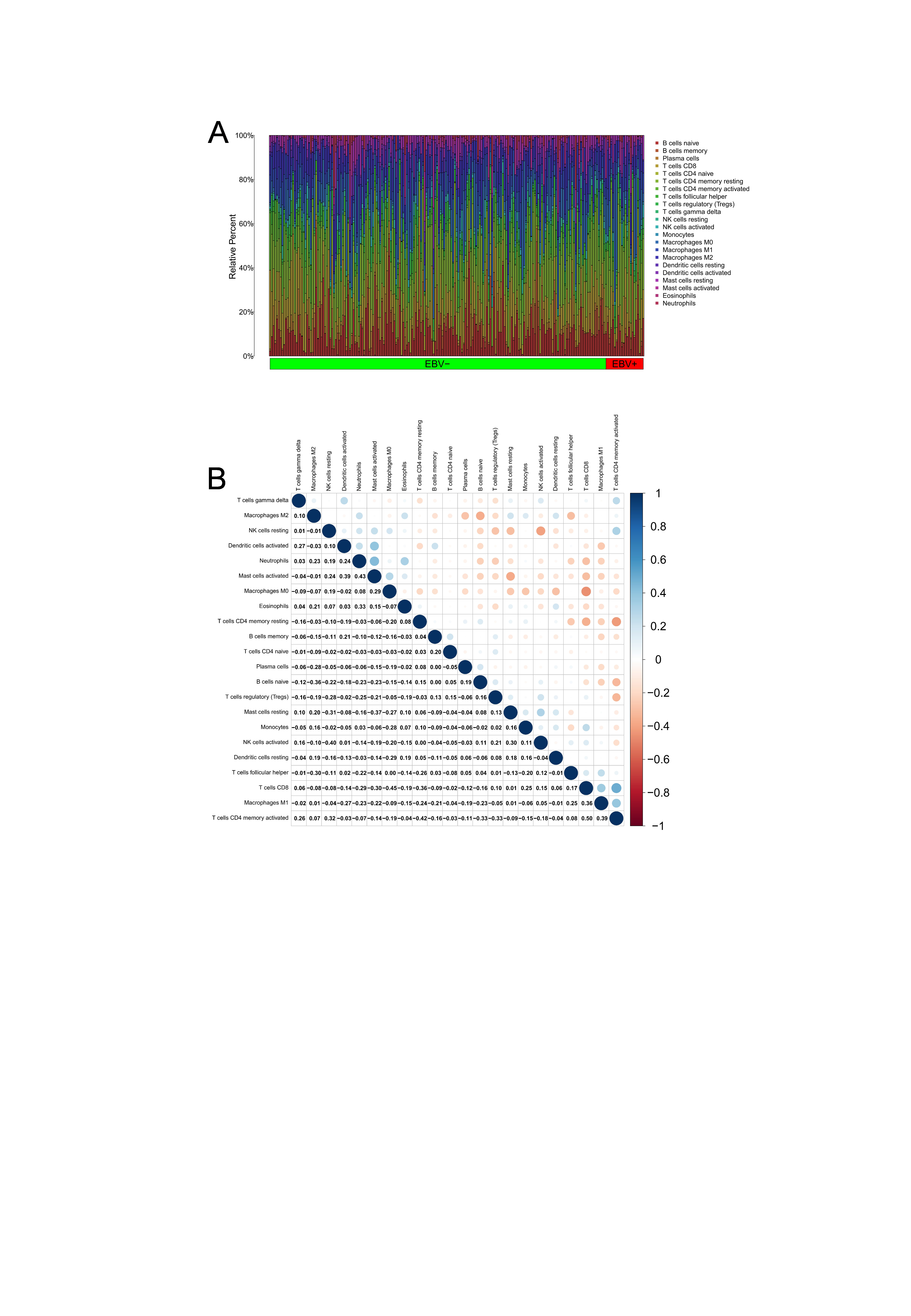

Supplement: Supplementary file 2 [file Image_1_v1.tif]

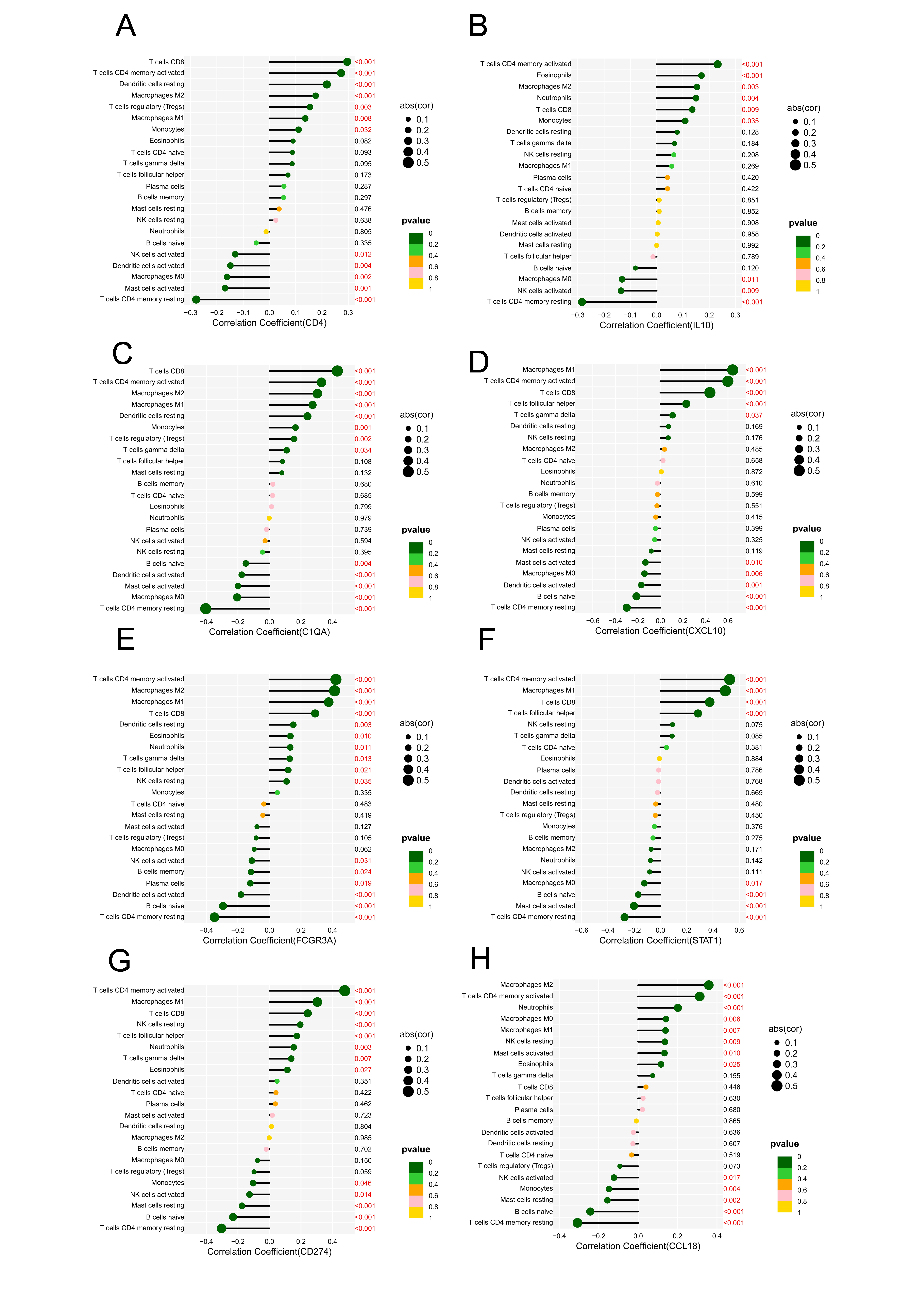

Supplement: Supplementary file 3 [file Image_2_v1.tif]
